# Factors influencing the use of contraceptives through the lens of teenage women: a qualitative study in Iran

**DOI:** 10.1186/s12889-018-5116-3

**Published:** 2018-01-30

**Authors:** Afrouz Mardi, Abbas Ebadi, Shirin Shahbazi, Sara Esmaelzade saeieh, Zahra Behboodi Moghadam

**Affiliations:** 1 School of Nursing and Midwifery, Tehran University of Medical Science, Tehran, Iran; 2 School of Health, Ardabil University of Medical Science, Ardabil, Iran; 3 Behavioral Sciences Research Center, Nursing Faculty of Baghiyatallah University of Medical Sciences, Tehran, Iran; 4 School of Nursing and Midwifery, Tehran University of Medical Science, Tehran, Iran; 5 School of Nursing and Midwifery, Alborz University of Medical Science, Alborz, Iran; 6 School of Nursing and Midwifery, Tehran University of Medical Science, Tohid Square, Eastern-Nosrat Street, Tehran, 1419733171 Iran

**Keywords:** Contraceptives, Teenage women, Qualitative study

## Abstract

**Background:**

One out of seven teenage girls in developing countries marries before the age of 15. While the fertility rate of teenage girls is high, the rate of contraceptive use remains low; therefore, this group of teenagers needs reproductive healthcare. This study was undertaken to explore factors influencing the use of contraceptives from the perspective of teenage women living in the city of Ardabil in Iran.

**Methods:**

This qualitative study was conducted with 14 married women aged 13–19 years who attended in urban-rural healthcare centers in Ardabil. Eligible women were recruited using purposive sampling and were invited to take part in individual in-depth semi-structured interviews. The duration of the interviews varied from 45 to 90 min with an average of 55 min. Sampling continued until data saturation was reached and no new data was collected. Each interview was tape-recorded after obtaining the participant’s permission, transcribed verbatim and analyzed for identifying categories and themes using conventional content analysis.

**Results:**

Three themes and eight subthemes were developed. The themes were as follows: “insufficient familiarity with contraceptive methods”, “pressure to become pregnant” and “misconceptions”.

**Conclusion:**

Despite the high prevalence of early marriage in Iranian society, teenage women are not empowered or prepared for marriage and birth control. Sexual and reproductive healthcare services to teenage women should be improved to meet their needs.

## Background

There are 580 million teenage girls worldwide and 88% live in low and middle income countries [1]. One out of seven girls in developing countries marries before the age of 15 [2]; hence, more than 39,000 girls become child brides every day worldwide [3].

Marriage patterns differ in Iran and regional countries and there continues to be pressure put on girls to marry [4]. In Iran, the legal minimum marriage age for girls is 13 years. More than 7.7% of girls living in Tehran marry before the age of 18. The rate of teenage marriage in rural areas is 19.6% [5]. According to the latest census, the highest rate of marriage was for the 20–24 year-old category for men and 15–19 year-old category for women [6]. Studies in Iran have shown that family structure, lack of autonomy in decision-making by teenage girls, the sexual, social and emotional needs of girls, low awareness of villagers and cultural poverty are the factors most strongly influencing early marriage. Villagers commonly believe that a girl should marry by the age of 15 or lose the opportunity of successful marriage. They believe that single girls have sexual or physical problems [7, 8]. In other words, early marriage and parenthood is encouraged and childbearing is very desirable, as the failure to bear a child would likely lead to remarriage of the husband with or without divorcing the first wife [9]. Children are considered to be treasures of the home and help maintain and strengthen the integrity of the family [10].

It has been reported that about 2.5 million girls under the age of 16 give birth in low-income countries annually [11]. In accordance with the Iranian Strategic Government Plan to increase the population in Iran that went into effect on 20 May 2014, the rate of population growth should increase in the next decade [12]. In addition, the fertility rate of young adults should increase considerably by 2025 [13].

While the average rate of marriage for girls under the age of 15 years in most areas of Iran is 5%, this rate is higher in provinces such as Ardabil, at about 9% [14]. Ardabil province is located in northwestern Iran in the region of Azerbaijan. The majority of its residents are Azeri and speak a Azeri-Turkish dialect [15]. The early age of marriage (under 15 years of age) is one of the greatest challenges to women’s health in this province [14].

The consequences of the early marriage of girls, families, children and the community in terms of health, social and financial concerns have been previously studied [16]. As soon as a teenage girl is married, she must tolerate a great amount of pressure to prove her fertility and strengthen her position in the family through childbirth. Many teenagers are inclined to become pregnant immediately after marriage. Consequently, the responsibilities of pregnancy, motherhood and being a wife are imposed on teenage girls from a young age [16].

At times, contraceptive failure leads to pregnancy. While the fertility rate of teenage girls is high, the rate of contraceptive use has remained low, which makes these young women extremely vulnerable in terms of reproductive healthcare [17, 18]. Teenage mothers and their children are at high risk for infantile and childhood mortality and morbidity, abortion, stillbirth, low birth weight, congenital anomalies, pre-eclampsia, anemia, hemorrhage, fistulas and dystocia [16, 19]. The consequences of childbirth in girls under the age of 14 years are destructive and include puerperal endometritis, the possibility of episiotomy, postpartum hemorrhaging, operative vaginal delivery and anemia. In addition, the rate of maternal death in young girls is four times higher than for women aged 20 to 24 years [20]. The results of a survey of teenagers in southeastern Iran showed that preterm delivery, intra uterine growth retardation (IUGR) and placenta previa were higher in teenagers than in older mothers, despite improved prenatal care [21].

It has been reported that the use of contraceptives can reduce the maternal mortality rate by 44% [22]. The use of contraceptives helps reduce unplanned pregnancies and subsequent unsafe and illegal abortions [23]. The use of contraceptives or the prevention of teen-pregnancy is a major step toward achieving sustainable development goals, including healthy lives and the promotion of well-being for people of all ages, gender equality and empowerment of women, high quality education to all and the eradication of poverty [24–27].

Many women rely on myths and misconceptions about the side effects and negative consequences of contraceptive methods and this lack of knowledge is associated with the poor use of contraceptives [28, 29]. Furthermore, the patterns of contraceptive use differs significantly between adolescent girls and adult women. Adolescents are less likely to use contraceptive methods than adult women. This difference is most likely due to insufficient knowledge and/or experience with regard to contraceptives and a lack of autonomy in decision-making by teenage women [18]. Moreover, the low educational levels and socio-cultural issues hinder their access to family planning methods immediately after marriage and limits the use of contraceptives in this age group [30].

Despite remarkable progress in maternal and child health in Iran, contraception and pregnancy in teenagers remain major problems in Iranian society [9]. Most studies conducted on teenagers in Iran have recruited unmarried girls who either are or are not sexually active. The needs, behaviors, practices, sexual health and use of contraceptives are quite different among teenagers who have married and live with their spouses and children. The results of studies carried out in other countries also cannot be generalized to the Iranian cultural context because of cultural differences. Some qualitative studies, however, have been conducted to improve understanding of the causes for the high rate of teenage pregnancies, such as the experiences of teenage women with the use of contraceptives.

The first author of this article was a resident of Ardabil province with over 20 years of educational and practical clinical experience in maternal and child health care. Because early marriage and pregnancy is prevalent in Ardabil [14], this study, which is part of a PhD dissertation, was conducted to explore factors influencing the use of contraceptives from the perspective of teenage women in Iran and in particular in Ardabil.

## Methods

### Design

This was a qualitative study using a conventional content analysis conducted by the first author. Content analysis is a qualitative descriptive method for the interpretation and classification of textual data through consideration of cultural and contextual aspects influencing the study phenomenon. The final products of data analysis are categories and themes [31].

### Participants

For the purpose of this study, 14 teenage married women were purposively sampled with maximum variation at urban-rural healthcare centers in Ardabil, Iran. For this purpose, the first author approached eligible participants. Each woman who met the inclusion criteria was provided with information about the study and encouraged to participate. The inclusion criteria were being a married woman of 13 to 19 years of age. In order to gain a variety of viewpoints, the women interviewed were of diverse demographic backgrounds in terms of age (early, mid and late adolescence), age at marriage, duration of marriage, urban or rural residence, educational level, parity and contraceptive method.

Although purposive criteria was applied, this was a convenience sample of whichever woman was asked and who agreed to participate in the study. In this group, the mean (±SD) age at marriage was 13.2 (±1.25) years and of the duration of marriage was 10 (±1.79) months. All were housewives and a majority of them were at junior high school.

### Data collection

Data collection was conducted between May 2016 and Oct 2016. The interviewer was a PhD candidate in the field of sexual and reproductive healthcare and a faculty member in a medical sciences university in Iran. The interviewer had practical experience in qualitative research. The eligible women, who attended health centers to obtain health care services, were informed of the aim of the study and were invited to take part in in-depth semi-structured interviews. The interviews were conducted individually and face-to-face in a private room in the healthcare centers. The duration of the interview sessions varied from 45 to 90 min with an average of 55 min.

Sampling continued until data saturation was reached and no new data was obtained. The interviews were carried out in the Azeri-Turkish language and were then translated into Farsi and English for the purpose of publication of the findings. Each interview was tape-recorded with the participant’s permission, transcribed verbatim and then analyzed. The questions used in the interviews were as follows:

- Please tell me about your experience with marriage.
- Please tell me about your experience regarding fertility.
- Can you describe your experiences with contraceptive use?
- Can you tell me whether or not you intend to use contraception?

### Data analysis

The data was analyzed based on the method suggested by Graneheim and Landman [32]. After each interview, they were transcribed verbatim and were read several times to get a sense of the whole. The transcriptions were then divided into meaning units, which were condensed and labeled with codes. The codes were sorted and divided into subcategories and categories given their similarities and differences and the hidden contents were developed as themes.

MAXQDA10 software was used for data management. The first author performed the data analysis and the other co-authors supervised this process. Discussions were held by the research team members to resolve disagreements.

### Trustworthiness

Lincoln and Guba explained that credibility, reliability and data transmission abilities improved the accuracy of qualitative research [33]. In this study, purposeful sampling with maximum variation, immersion in the data, member checking and an audit trail helped validate the coding process and data analysis. Peer checking, long-term engagement with participants and maintaining ongoing relationships using notes and journals improved the depth of data analysis. Note that the translation of the interviews from Turkish to Farsi and English were performed by two translators separately and were ultimately checked by a third person.

### Ethical considerations

This study was part of the first author’s doctoral dissertation and was conducted with the approval of the Ethics Committee affiliated with Tehran University of Medical Sciences (decree number = IR.TUMS.REC.1395.2576). The participants were informed of the aim and method of the study and gave the permission to tape-record their voices. Before the interviews, all participants signed written informed consent forms. The participants were assured that the study results would be completely confidential and anonymous. They were informed of the voluntary nature of participation in this study. They were also given the right to refuse to participate in the interviews at any time without being penalized.

## Results

The mean age of the teenage women was 14.9 (±1.62) years. Of the participants, four were pregnant, three had no children, six had one child having a mean age of 7.25 (±3.48) months, and one had undergone an abortion for medical reasons. It was determined that five people of the non-pregnant women were not using any contraceptive method. In this respect, three women used the rhythm method, one women used oral contraceptive pills and one woman used condoms, (Table 1). Data analysis led to the development of three themes and eight subthemes. The themes were: “insufficient familiarity with contraceptive methods”, “pressure to become pregnant” and “misconceptions” (Table 2).

**Table 1:** Characteristics of participants

Characteristic	Number
Age	
13 > =	3
14–15	5
16–17	5
18–19	1
Mean	14.9
Age of marriage	
12	5
13	*4*
14	3
15	1
16	1
Mean	13.2
Length of marriage (month)	
6>	2
6–12	3
13–24	6
25–36	3
Mean	10
Educational level of participants	
Elementary school	4
Junior high school	7
Senior high school	3
Age of husband	
20–21	2
22–23	3
24–25	6
26–27	3
Mean	23.85
Contraceptive method (among non-pregnant women)	
Withdrawal	3
Condom	1
OCP	1
Nothing	5
Having child	
0	3
1	6
Pregnant	4
Abortion previous	1

**Table 2:** Themes and subthemes

Insufficient familiarity with contraceptive methods	Low quality of education and counseling in the healthcare system
	A Lack of education by the family
Pressure to become pregnant	Proving the identity
	Consolidating the position in the husband’s family
	Relieving loneliness
	A low decision-making power
Misconceptions	The fear of infertility
	The fear of possible side effects

## Insufficient familiarity with contraceptive methods

The teenage women’s awareness of contraceptive methods was very low because of the poor quality of educational services and consultation in the healthcare system. They also did not receive the required information from their parents.

### Low quality of education and counseling in healthcare system

Education about contraceptive methods was presented to couples on the day they registered to marry; however, lack of time, lack of concentration in the classes and lack mental readiness meant that the information presented was not useful. One young women referred to the healthcare system stated that no comprehensive education on the use of contraception was available because of the overcrowded conditions of the healthcare center.

A 13-year-old woman who had married 40 days earlier said about premarital counseling classes: “They said something that I cannot remember because I was preoccupied during those days. I was stressed out and did not know what I was doing. I was not relaxed. When I talked about it with my fiancée, I was worried. I could not comprehend anything at all.” (Participant 10).

A 15-year-old woman who was in her 28th week of pregnancy said: “In the healthcare center, I was told ‘try not to get pregnant for now’ , but they did not tell me how to do so.”

### Lack of education by family

Talking about sexual issues and contraceptive methods in the family were primarily considered to be taboo. If the couple needed information, embarrassment, fear of being subjected to negative attitudes and lack of knowledge of the mothers of the women prevented proper communication between the young women and family members. A 15-year-old woman who recently had a miscarriage declared: “Before marriage, I was not taught anything about it [contraception]. At the beginning of my marriage, I asked my older sister some questions about it, but she said: ‘you are too young’. She was embarrassed to discuss such things.” (P 8).

## Pressure to become pregnant

Most of the teenage women intended to become pregnant soon after marriage. Even if they personally wanted to delay motherhood in order to study or work or for other reasons, socio-cultural pressures did not allow them to do so.

### Proving identity

Many of the teenage women try to prove their identity as adult women by becoming pregnant at an early age. Pregnancy signified health and fertility. In fact, Iranian society infuses a sense of confidence and self-esteem in pregnant women.

One participant said: “I was happy and thought that I had become an adult and was like my mother. I could conceive a child and grow it.” (P 13).

Another participant stated: “In my village, once a girl is married, she should have a child. If not, others will think that she is not healthy.” (P 10).

### Consolidating one’s position in the husband’s family

Pregnancy for a young woman guaranteed security and comfort in the family.

A woman who does not succeed in giving birth to a child can be abused and neglected and even the continuation of her marital life can be compromised. For the women, pregnancy achieves a with a sense of power and peace of mind connected to their husband’s satisfaction and marital stability.

One participant said: “When the pregnancy test was positive, my husband promised me not to mistreat me again during our life and to meet my all needs. He had mistreated me before that.” (P 1).

A 14-year-old woman who married despite her family’s opposition said: “I was scared. If I did not get pregnant, my husband’s family would force my husband to marry another girl, even if he did not divorce me. Also, I thought that perhaps my husband’s family members would love me for the sake of the child.” (P 13).

### Relieving loneliness

The findings of this study showed that the majority of the young women decided to immediately become pregnant because they had been forced to drop out of school, limit their social movement and stay at home. Having a child was a way to escape loneliness and providing a companion at home. Having a child*,* looking after it and providing for it gave new meaning and significance to life and brought hope and prosperity to marital life.

One of the participants stated: “I was alone so much. I had no relatives to talk to and was not allowed to leave the house. It is traditional here for a bride not to leave the house without her husband. I wanted a child to have someone to talk to.” (P 9).

Another participant who had experienced an unwanted pregnancy said: “I did not want to get pregnant so early. I had told this to my husband. When I found out I was pregnant, I wanted to have an abortion, but my husband said, ‘You are alone at home. Let’s have the child and I will help you with it.’” (P 3).

### Lack of decision-making power

Almost all of the women lacked the power to make decisions because they were young and had no status in the family. They were unable to decide about the use of contraceptive methods. All decisions about childbearing, including the time, interval and number of children, were made by their husbands and family members. Some may suffer repeated pregnancies to finally give birth to a baby boy, because of the husband’s desire to have a son.

A 14-year-old participant who was in her 28th week of pregnancy and had been told that her child was a girl according to para-clinic tests said: “I don’t know. My husband should want it. I have no choice and if he wants a child, he wants it to be a boy. If he does not want to prevent pregnancy, I can do nothing.” (P 13).

Another participant answered the question of ‘Would you like to have a child?’ as follows: “I do not know. I should find out what the others [the husband and his family members] want.” (P 11).

## Misconceptions

This theme was consisted of two subthemes: “fear of infertility” and “fear of possible side effects”. Many of the women had no inclination to use contraceptive methods due to misconceptions and inappropriate information provided by friends and relatives. Fear and concern were major barriers to the use of contraceptives. The most important concern mentioned by the participants was the fear of infertility caused by a particular method. In many cases, this was the main reason preventing them from using contraception.

### Fear of infertility

Some participants believed that contraceptive methods led to infertility. A 16-year-old woman who became pregnant after 1 year of marriage said: “My mother told the relatives to ‘advise her to do not use contraceptives so she will not become pregnant in the future’”. (P 5).

### Fear of possible side effects

One reason not to use contraceptives was fear of possible side effects. One participant, in response to the question “What do you know about contraceptive methods?” said: “I know nothing. I only know that they are harmful. For instance, I have heard that oral contraceptives may lead to the development of cysts.” (P 4).

## Discussion

This is the first qualitative study specifically aimed at exploring the factors influencing the use of contraceptives among teenage women in an Iranian setting**.** The study results reveal that, although most participants intended to postpone their pregnancy and motherhood, they had little inclination to use contraceptives. Factors such as insufficient familiarity with contraceptive methods, pressure to become pregnant and misconceptions affected their decisions not to use contraceptives.

The results show that the teenage women did not receive appropriate information about contraception from their family or the healthcare system. Other studies have reported that most teens have little awareness about their sexual and reproductive health and what knowledge they do have is incomplete and unreliable [34]. The reason for this could be that families and educational institutions do not empower and prepare girls for marriage and the acceptance of marital responsibilities such as family planning [35].

In the current study, most participants were dissatisfied with the time and quality of family planning education at pre-marital or healthcare centers and considered their training unhelpful. In addition, they had no appropriate access to relevant information from parents. It appears that this issue is more common among the Azeri people than in other regions of Iran and is cultural and religious in origin. A number of Iranian studies have confirmed these results and have shown that parents do not talk about sexual matters with their children. There is no appropriate interaction between mothers and daughters out of embarrassment, fear of being subjected to negative attitudes and lack of knowledge on the part of the mothers. Adolescents are also embarrassed to talk about sexual issues and taboos, beliefs and traditions also hinder young adolescent access to this required information [34–36].

Pressure to become pregnant was another finding of this study. The participants viewed pregnancy as a way to prove their identities and consolidate their position in the husband’s family. Nearly all of them had low autonomy in decision-making with regard to childbearing and the majority of teenage mothers decided on early pregnancy in order to escape from loneliness.

According to the literature, about 20% of young wives in developing countries (except China) attempt to have a child under the age of 18 [37] because becoming a mother is an important aspect of life and motherhood is considered to be the identity of a woman [38]. In the current study, it was found that teenage women thought that pregnancy would prove their identity and fertility. If a woman’s baby was a boy, she would become more popular in her husband’s family. This is in compliance with findings of a study in Mozambique which showed that having a child makes parents proud and helped them preserve their identities as males and females [38]. Another study in Iran showed that pregnancy makes young women feel that they have appropriately developed and are able to bear a child [13]. The social value of a teenage women increases when she bears a child, especially a boy [17]. In Iran, some families insist on having a male heir in the family; therefore, women in such families are forced to submit to repeated pregnancy in order to finally give birth to a male child [39].

The study results indicate that another reason for the pressure for immediate pregnancy was a woman’s limited decision-making power. The participants were not mature enough and had insufficient autonomy when it came to decision-making in married life. The study participants were too young to have learned essential life skills and were not empowered to make proper decisions about their reproductive health. Most were not able to anticipate the consequences of early pregnancy and motherhood and had insufficient autonomy in decision-making at home. They thus allowed others to decide for them on the use of contraception.

These findings are supported by those of other studies that report the decision to start or stop taking contraceptives is not primarily in the woman’s hands, but is influenced by others from their social network [28, 40]. While most modern and traditional contraceptive devices are designed for women, it is traditionally the men who decide what contraceptives to use [38]. Sharma stated that social pressures about the “ideal woman” and proving fertility along with poor communication skills reduce a woman’s decision-making power about sexual and reproductive health [41].

The desire to escape loneliness and have a companion was another reason for not using contraceptives in this study. Teen mothers believed a child could distract and amuse them. It appears that this is a recent development among Iranian teenage. They experience isolation and loneliness at home after they are deprived of educational and social activities.

Beliefs about contraceptive methods tended to be in two forms: fear of possible side effects from contraceptives and fear of infertility. Fear was a major obstacle to the use of contraceptive methods and stemmed from inappropriate information gained from friends and relatives. The biggest concern for most participants was fear of infertility and this hindered the use of effective contraception. Many of these women had no inclination to use contraceptives because they feared the possible side effects, while they knew nothing about the risks of pregnancy for women under the age of 18 years. These results were in line with the results of similar studies [18, 28, 42].

Studies have reported that exaggerated beliefs about the side effects of contraceptive methods are rooted in myth and misconceptions [28, 42, 43]. Myths and rumors narrated by peers and partners affect the use of contraception [28, 44]. In addition, those who want to delay pregnancy preferred traditional methods to modern methods from fear of infertility [23]. In Malawi, it was found that 40% of 19–12 year-old girls believed that if they had sexual relationships in a standing position, the probability of pregnancy would be low. More attention must be devoted to the education and health of teenage married women to reduce the consequences of early and high-risk pregnancies in this vulnerable group.

## Limitations

Translation of the interviews from Turkish to Farsi and English were a limitation of the study. These were performed separately by two proficient translators and were ultimately checked by a third person. Furthermore, this study was conducted on a limited number of teenage women in one city in Iran; therefore, the results could not necessarily be generalized to all Iranian teenage women.

## Suggestions for future studies

This population is very vulnerable and much more qualitative research is required to extend the insights and findings of this study to different parts of Iran and other developing countries. In addition, this study focused on women; therefore, it is recommended that future studies also consider the men experiences regarding contraceptive methods, because they have a significant effect on the demand and use of contraceptives.

## Conclusion

The findings of the current study show that the majority of teenage women were reluctant to use contraceptive methods because they are insufficiently familiar with contraceptive methods, are under pressure to get pregnant and because they harbor misconceptions about contraceptives.

The poor quality of education offered by health practitioners and parents was an important factor limiting the use of contraceptive methods. Teenage women had little knowledge about contraceptives and little autonomy in decision-making about pregnancy. They felt that child-bearing proved their identities and consolidated their positions in their married life and provided an escape from loneliness. Of course, in these cases, incorrect beliefs had a significant impact.

These findings argue the need to develop culturally-sensitive programs and interventions that address the needs of this vulnerable group. This includes training for effective and friendly interaction between mothers and girls and special education for health practitioners. There is also a need for intersectional collaboration between policymakers, cultural and religious organizations and NGOs to improve the sexual and reproductive health of adolescent women.

## Abbriviations

IUGR: Intra uterine growth retardation
MAXQDA: Qualitative data analysis software and Mac OS X
NGO: Non-governmental organization
SD: Standard deviation
